# Joint association of physical function and sleep duration with incident depressive symptoms among middle-aged and older Chinese

**DOI:** 10.1186/s12889-026-26677-x

**Published:** 2026-02-21

**Authors:** Quan Zhou, Fanhao Meng

**Affiliations:** 1 Department of Social Work and Social Administration, The University of Hong Kong, Hong Kong, Pokfulam China; 2 Department of Psychiatry, Huzhou Third Municipal Hospital Affiliated to Huzhou University, Huzhou, 313000 China

**Keywords:** Sleep duration, Physical dysfunction, Depressive symptoms, CHARLS, Cohort study

## Abstract

**Objective:**

To explore the independent, joint, interactive, and mediating effects of physical function (PF) and sleep duration (SD) on incident depressive symptoms (IDS) among Chinese adults aged ≥ 45 years, and to recommend appropriate PF-SD combination strategy to lower IDS risk.

**Methods:**

Data were obtained from the China Health and Retirement Longitudinal Study (CHARLS), with a nine-year follow-up from 2011 to 2020. Physical function was assessed with a 9-item questionnaire, SD was self-reported, and IDS was defined by the Center for Epidemiological Studies Depression Scale ( CES-D). Cox regression, restricted cubic spline (RCS) model, Subgroup and sensitivity analysis, interaction, and mediation analyses were applied.

**Results:**

Among 6,189 community-dwelling adults (mean age 56.6 ± 7.9 years; 52.4% male), 1,815 incident IDS events (29.3%) were documented over 9 years of follow-up. Both physical dysfunction (PD) and short sleep duration (SSD) were independently associated with IDS (PD: HR = 1.37; SSD: HR = 1.15–1.34; all P < 0.05). The SSD–IDS association remained robust across PD and non-physical dysfunction (NPD) subgroups (HR = 1.13–1.38; all P < 0.05 except for midday nap duration [MND] < 30 min in NPD). Relative to the PD + SSD referent, all other combinations exhibited significantly lower hazard ratios (HR = 0.53–0.85; all P < 0.05). Restricted cubic spline analyses revealed non-linear relationships of total sleep duration (TSD) and nighttime sleep duration (NSD) with IDS (*P* for non-linearity = 0.002), with no further risk reduction beyond TSD > 8 h or NSD > 7.5 h (both *P* > 0.05). Mediation analysis indicated that TSD and NSD accounted for 3.55% and 4.0%, respectively, of the PD–IDS effect.

**Conclusion:**

PD and SSD are independent risk factors for IDS among middle-aged and older Chinese, with PD + SSD combination conferring the highest risk. Recommended risk-mitigation strategies are: for NPD individuals, NSD ≥ 6 h; for PD individuals, NSD ≥ 6 h or MND ≥ 30 min. TSD and NSD partially mediate the PD-IDS relationship. Long sleep duration did not increase the risk of IDS.

**Graphical Abstract:**

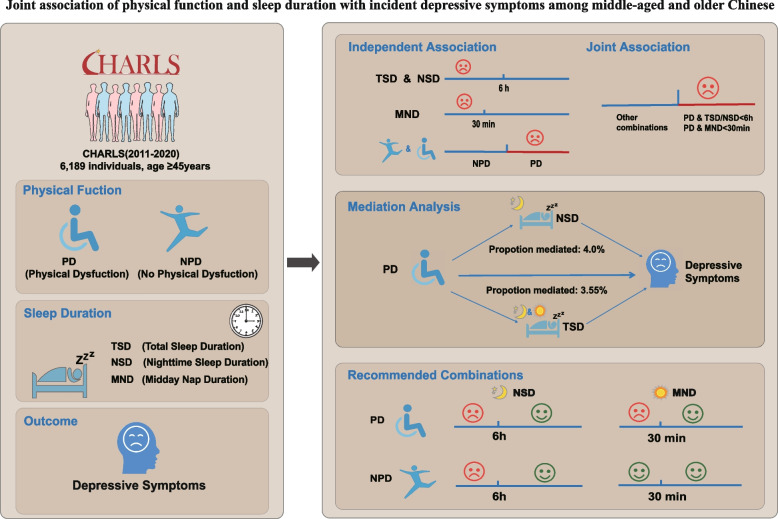

**Supplementary Information:**

The online version contains supplementary material available at 10.1186/s12889-026-26677-x.

## Introduction

Depressive disorders have been reported by the World Health Organization as one of the leading causes of disability worldwide and a major contributor to the global burden of disease, affecting more than 350 million people [1, 2] In China, approximately 41.01 million individuals are estimated to be living with depressive disorders, ranking second globally in terms of the affected population size [3]. By 2030, depressive disorders are projected to become the leading cause of global disease burden [4]. With the continuing growth of the aging population, health concerns among middle-aged and older adults have attracted increasing attention [5]. The prevalence of depression among older adults exceeds 20%, and a substantial proportion present with depressive symptoms that do not meet clinical diagnostic criteria [6, 7]. Existing evidence further suggests that the prevalence of depressive symptoms (DS) among middle-aged and older adults in China has risen to 37.62% [8], imposing a considerable burden on families and society. Moreover, depression may lead to reduced physical activity, diminished quality of life, and even self-harm or suicide [9], underscoring its importance as a major public health challenge globally and in China.

Sleep duration (SD) is a potentially modifiable behavioral factor associated with depression. Evidence indicates that short sleep duration (SSD) is associated with an increased risk of depression onset [10] and recurrence [11]. However, the association between long sleep duration (LSD) and incident depressive symptoms (IDS) remains controversial [12, 13]. Meanwhile, Previous research on physical function (PF) and depression has predominantly focused on single indicators such as grip strength or gait speed, or has examined the association between overt functional limitations or disability—such as impairments in instrumental activities of daily living (IADL) and activities of daily living (ADL)—and depressive outcomes [14–17]. A recent study reported that PF encompassing upper-limb, lower-limb, and trunk functional capacities, as well as ADL, was significantly associated with DS among middle-aged and older adults in China [18]. Nevertheless, evidence remains limited in community-dwelling middle-aged and older populations regarding how PF and multidimensional SD—nighttime sleep duration (NSD), midday nap duration (MND), and total sleep duration (TSD)—jointly relate to IDS.

From a public health perspective, PF and SD may be interrelated and may jointly influence incident depressive symptoms (IDS) through partially overlapping pathways [19, 20]. Notably, declines in PF often precede overt ADL/IADL disability and may be accompanied by activity restriction, increased fatigue or pain, and reduced social participation, thereby facilitating the development of unfavorable sleep patterns [18, 21, 22]. In addition, prior studies have suggested that, among older adults, physical activity levels and sleep duration are significantly associated with cognitive status [23, 24], further underscoring the importance of functional status and sleep characteristics for health outcomes and their potential synergistic effects. Accordingly, clarifying the independent and joint associations of PF and multidimensional SD with IDS, and further examining whether SD modifies the PF–IDS association (effect modification) or serves as a potential pathway linking PF to IDS (mediation), may help identify more targeted high-risk profiles and provide a stronger evidence base for early prevention strategies in the context of population aging.

To address these gaps, we conducted a prospective cohort study using data from the China Health and Retirement Longitudinal Study (CHARLS). The objectives were threefold. First, we evaluated the independent and joint associations of objectively assessed PF—a composite measure integrating upper-limb, lower-limb, and trunk functional capacity and strength—and SD with the risk of IDS. SD was categorized into short, mediate, and long groups as follows: TSD (< 6 h, 6–8 h, ≥ 8 h), NSD (< 6 h, 6–8 h, ≥ 8 h), and MND (< 30 min, 30–90 min, ≥ 90 min). Second, guided by a prespecified conceptual framework, we examined whether SD modifies the association between PF and IDS (effect modification/interaction) and whether SD may partially explain the PF–IDS association (a potential pathway). Third, we sought to identify PF-SD combinations associated with a lower risk of IDS, thereby providing evidence to inform targeted prevention strategies for middle-aged and older adults in China.

## Methods

### Study design and participants

This prospective cohort study leveraged data from the China Health and Retirement Longitudinal Study (CHARLS) 2011 baseline, 2013, 2015, 2018 and 2020 follow-up waves. CHARLS, implemented by the National School of Development at Peking University, is an ongoing household-based panel survey of community-dwelling adults aged ≥ 45 years. The 2011 baseline successfully enrolled 17,705 participants. The protocol was approved by the Biomedical Ethics Committee of Peking University (IRB00001052-11015) and adhered to the Helsinki Declaration guidelines. All respondents provided written informed consent. Further details are available at http://charls.pku.edu.cn.

**Fig. 1:**
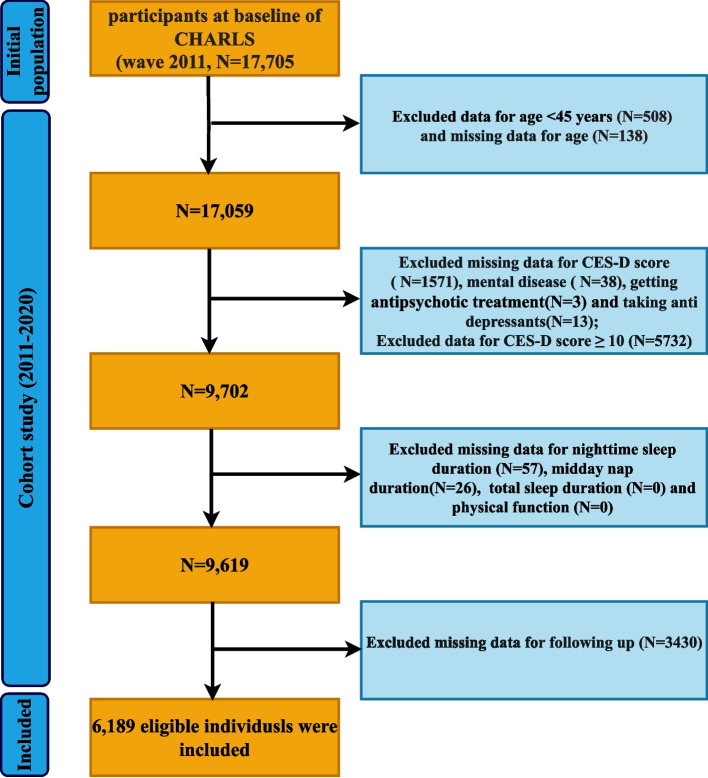
Flow diagram of the screening and enrollment of study participants

Participants were selected according to the following prespecified inclusion and exclusion criteria: (1) age < 45 years (*n* = 508) or missing age data (*n* = 138); (2) In 2011, self-reported physician-diagnosed psychiatric disorders (*n* = 38), current got antipsychotic treatment (*n* = 3), use of antidepressants (*n* = 13), missing data for Center for Epidemiologic Studies Depression Scale (CES-D) scores (n = 1,571), or CES-D ≥ 10 (*n* = 5,732); (3) missing data for physical dysfunction (PD) and SD (*n* = 83); (4) loss to follow-up (*n* = 3,430). After sequential application of these criteria, 6,189 participants remained for the final analytic sample (Fig. 1).

### Assessment of Physical Function

The CHARLS questionnaire includes an evaluation of nine activities related to physical functioning. These activities comprise walking 100 m, walking one kilometer, jogging or running one kilometer, rising from a chair after prolonged sitting, repeatedly climbing several flights of stairs, bending, kneeling, or crouching, extending arms to shoulder height, lifting or carrying objects heavier than 5 kg, and picking up a small coin from a table. Each item is scored using a four-level response scale: (1) No difficulty; (2) Some difficulty but can be done independently; (3) Much difficulty and requires assistance; (4) Unable to perform. In this research, participants reporting any degree of difficulty on one or more items were classified as having PD. Those reporting no difficulty across all items were defined as having NPD [18].

### Assessment of Sleep Duration

Three sleep duration variables were examined: NSD, MND, and TSD. NSD was assessed by asking, “During the past month, on average how many hours of actual sleep did you get each night? This may be shorter than the time spent in bed.” MND was measured with the question: “During the past month, how long did you usually nap after lunch (average minutes per nap)?” TSD was calculated as the sum of NSD and MND. Based on prior studies, both NSD and TSD were categorized into three groups: SSD (< 6 h), moderate sleep duration (MSD, 6–8 h), and LSD (> 8 h) [14, 25]. Similarly, MND was grouped as SSD (0–30 min), MSD (30–90 min), and LSD (> 90 min) [26].

### Assessment of Depressive Symptoms

Depressive symptoms were measured using the 10-item Center for Epidemiological Studies Depression Scale (CES-D). This instrument evaluates mood and behavioral patterns associated with depressive symptoms experienced during the previous week and has demonstrated good reliability and validity in Chinese populations [27]. Each item is rated on a 4-point scale ranging from 0 (rarely or never) to 3 (most or all of the time). Total scores range from 0 to 30, with higher scores indicating more severe depressive symptoms. Following previously established criteria [13], participants with a total score of 10 or higher were classified as having depressive symptoms in this study.

### Covariates

At baseline (2011), trained interviewers collected information on sociodemographic characteristics and health-related factors using a structured questionnaire. Sociodemographic data included age, sex (male, female), marital status (married, unmarried), residence (urban, rural), and education level (below primary, primary, junior high, senior high or above). Health-related factors covered current smoking status (non-smoker, smoker) and alcohol consumption (non-drinker, drinker). Anthropometric measures comprised BMI (kg/m^2^) and waist circumference (cm). Self-reported chronic conditions included hypertension, heart disease, stroke, and lung disease. Covariate selection was based on existing literature, social and clinical relevance. Those covariates altering initial regression coefficients by more than 10% were also included.

### Statistical analysis

All normally distributed continuous variables were reported as mean ± standard deviation (SD), while those that were skewed were expressed as median with interquartile range (IQR). Categorical variables were presented as percentages. To compare continuous variables across different groups, the independent samples Student’s t-test was applied for normally distributed data, whereas the Mann–Whitney U test was utilized for non-normally distributed data. Categorical variables were analyzed using the chi-square test when appropriate.

Multivariable Cox proportional-hazards models were used to estimate the independent and joint associations of PD, TSD, NSD and MND with IDS. Three models were built: Model 1 was modified for sex and age; the second model further adjusted for marital status, residence, educational level, alcohol consumption, smoking status, body mass index and waist circumference; Model 3 further adjusted for hypertension, heart disease, stroke and lung disease. A restricted cubic spline (RCS) function was incorporated into Cox regression Model 3, with four knots placed at the 5th, 35th, 65th, and 95th percentiles of the exposure distribution, to flexibly model the dose–response relationship. For nonlinear associations, piecewise regression models were applied.

Subgroup analyses were performed with stratified Cox proportional hazards models. Interaction among subgroups was tested with the likelihood-ratio test. Sensitivity analyses repeated multivariable Cox regression models.

Mediation analysis, with bootstrap testing, treated IDS as the outcome, PD as exposure, and TSD/NSD/MND as mediators (or vice versa), adjusting for covariates. Additive and multiplicative interactions between SSD/LSD, PD and IDS were evaluated.

Missing covariates were multiply imputed (five imputations, chained equations, R MI procedure); missing data on population, exposure or outcome were excluded during screening.

All statistical analyses were conducted using R Statistical Software (Version 4.2.2, http://www.R-project.org, The R Foundation) and Free Statistics Analysis Platform (Version 2.2, Beijing, China, http://www.clinicalscientists.cn/freestatistics).

## Result

### Baseline characteristics

A total of 6,189 participants were enrolled and divided into the PD (*n* = 3,223) and NPD (*n* = 2,966) groups (Table 1). The PD group was older (57.9 ± 8.2 vs. 55.2 ± 7.2 years), included more women (55.9% vs. 38.5%), had a higher proportion of rural residents (62.1%), and showed lower educational attainment (65.6% with primary school or less). Smoking (28.3% vs. 37.8%) and alcohol consumption (31.2% vs. 43.9%) were less prevalent in the PD group. Hypertension (45.5%), heart disease (11.9%), and other comorbidities were significantly more common in the PD group. TSD (7.2 ± 1.8 h) and NSD(6.6 ± 1.6 h) were shorter in the PD group (above all *P* < 0.05), whereas midday nap duration did not differ between groups (*P* = 0.813).

**Table 1:** Baseline characteristics of the study population

Variables	Total (*n* = 6189)	NPD (*n* = 2966)	PD (*n* = 3223)	*P* value
Age, years	56.6 ± 7.9	55.2 ± 7.2	57.9 ± 8.2	< 0.001
Sex, n (%)				< 0.001
Female	2944 (47.6)	1143 (38.5)	1801 (55.9)	
Male	3245 (52.4)	1823 (61.5)	1422 (44.1)	
Marry, n (%)				< 0.001
Unmarried	425 (6.9)	160 (5.4)	265 (8.2)	
Married	5764 (93.1)	2806 (94.6)	2958 (91.8)	
Rural, n (%)				< 0.001
Urban	2551 (41.2)	1331 (44.9)	1220 (37.9)	
Rural	3638 (58.8)	1635 (55.1)	2003 (62.1)	
Education level, n (%)				< 0.001
Below primary school	2166 (35.0)	799 (27)	1367 (42.5)	
Primary school	1383 (22.4)	639 (21.6)	744 (23.1)	
Middle school	1627 (26.3)	899 (30.3)	728 (22.6)	
High school or higher	1007 (16.3)	626 (21.1)	381 (11.8)	
Smoking status, n (%)				< 0.001
No	4158 (67.2)	1846 (62.2)	2312 (71.7)	
Yes	2031 (32.8)	1120 (37.8)	911 (28.3)	
Alcohol consumption, n (%)				< 0.001
No	3884 (62.8)	1665 (56.1)	2219 (68.8)	
Yes	2305 (37.2)	1301 (43.9)	1004 (31.2)	
BMI, kg/m^2^	23.6 (21.4, 26.1)	23.5 (21.4, 26.0)	23.8 (21.5, 26.6)	0.007
Waist circumference, cm	84.7 ± 13.4	84.1 ± 12.0	85.2 ± 14.6	0.003
Hypertension, n (%)				< 0.001
No	3601 (58.2)	1846 (62.2)	1755 (54.5)	
Yes	2588 (41.8)	1120 (37.8)	1468 (45.5)	
Heart disease, n (%)				< 0.001
No	5619 (91.2)	2798 (94.5)	2821 (88.1)	
Yes	543 (8.8)	162 (5.5)	381 (11.9)	
Stroke, n (%)				< 0.001
No	6108 (98.7)	2947 (99.4)	3161 (98.1)	
Yes	81 (1.3)	19 (0.6)	62 (1.9)	
Lung disease, n (%)				< 0.001
No	5791 (93.6)	2843 (95.9)	2948 (91.5)	
Yes	398 (6.4)	123 (4.1)	275 (8.5)	
TSD, hours	7.3 ± 1.8	7.4 ± 1.7	7.2 ± 1.8	< 0.001
NSD, hours	6.7 ± 1.6	6.9 ± 1.5	6.6 ± 1.6	< 0.001
MND, minutes	2.0 (0.0, 60.0)	1.5 (0.0, 60.0)	2.0 (0.0, 60.0)	0.813
Depressive symptoms, n (%)				< 0.001
No	4374 (70.7)	2263 (76.3)	2111 (65.5)	
Yes	1815 (29.3)	703 (23.7)	1112 (34.5)	

Continuous variables are presented as mean ± SD or median (25th, 75th percentile), and categorical variables are presented as number (percentage)

*PD* physical dysfunction, *NPD* no physical dysfunction, *BMI* body mass index, *TSD* total sleep duration, *NSD* nighttime sleep duration, *MND* midday nap duration

### Independent association of PD and SD with DS

In the multivariable Cox analysis from Table 2, after full adjustment, PD was positively correlated with the risk of IDS (HR = 1.37, 95%CI:1.24–1.51, *P* < 0.001). TSD, NSD and MND as continuous variables showed inverse associations with risk (TSD, HR = 0.93, 95%CI:0.90–0.95; NSD, HR = 0.93, 95%CI:0.90–0.96; MND, HR = 0.98, 95%CI:0.97–0.99, all *P* < 0.05). Compared with moderate sleep duration (MSD), SSD significantly elevated risk (TSD HR = 1.34, 95%CI:1.17–1.54; NSD HR = 1.31, 95%CI:1.16–1.49; MND, HR = 1.15, 95%CI:1.03–1.29, all *P* < 0.05), while LSD had no significant association (*P* > 0.05).

**Table 2:** Multivariable Cox analysis of PD, TSD, NSD, and MND with IDS

Variable	Number		Unadjusted		Model1^a^		Model2^b^		Model3^c^	
	**Total**	**Event(%)**	**HR(95%CI)**	***P* value**	**HR(95%CI)**	***P* value**	**HR(95%CI)**	***P* value**	**HR(95%CI)**	***P* value**
PD										
No	2966	703 (23.7)	1(Ref)		1(Ref)		1(Ref)		1(Ref)	
Yes	3223	1112 (34.5)	1.67 (1.52 ~ 1.83)	< 0.001	1.51 (1.37 ~ 1.66)	< 0.001	1.4 (1.27 ~ 1.55)	< 0.001	1.37 (1.24 ~ 1.51)	< 0.001
TSD	6189	1815 (29.3)	0.91 (0.89 ~ 0.93)	< 0.001	0.92 (0.9 ~ 0.95)	< 0.001	0.93 (0.9 ~ 0.95)	< 0.001	0.93 (0.9 ~ 0.95)	< 0.001
6–8 h	2434	690 (28.3)	1(Ref)		1(Ref)		1(Ref)		1(Ref)	
< 6 h	932	351 (37.7)	1.53 (1.35 ~ 1.74)	< 0.001	1.45 (1.27 ~ 1.65)	< 0.001	1.35 (1.17 ~ 1.54)	< 0.001	1.34 (1.17 ~ 1.54)	< 0.001
≥ 8 h	2823	774 (27.4)	0.94 (0.85 ~ 1.04)	0.254	0.95 (0.86 ~ 1.06)	0.361	0.9 (0.81 ~ 1.01)	0.071	0.9 (0.81 ~ 1.01)	0.076
*P* for trend				0.179		0.288		0.052		0.057
NSD	6189	1815 (29.3)	0.92 (0.89 ~ 0.95)	< 0.001	0.93 (0.9 ~ 0.96)	< 0.001	0.93 (0.9 ~ 0.96)	< 0.001	0.93 (0.9 ~ 0.96)	< 0.001
6–8 h	2886	801 (27.8)	1(Ref)		1(Ref)		1(Ref)		1(Ref)	
< 6 h	1190	415 (34.9)	1.46 (1.3 ~ 1.65)	< 0.001	1.41 (1.25 ~ 1.59)	< 0.001	1.32 (1.17 ~ 1.5)	< 0.001	1.31 (1.16 ~ 1.49)	< 0.001
≥ 8 h	2113	599 (28.3)	1.05 (0.95 ~ 1.17)	0.358	1.05 (0.95 ~ 1.17)	0.328	0.99 (0.89 ~ 1.11)	0.917	0.99 (0.89 ~ 1.11)	0.903
*P* for trend				0.181		0.17		0.857		0.876
MND	6189	1815 (29.3)	0.97 (0.96 ~ 0.98)	< 0.001	0.98 (0.97 ~ 0.99)	< 0.001	0.98 (0.97 ~ 0.99)	0.001	0.98 (0.97 ~ 0.99)	0.001
30–90 min	1967	543 (27.6)	1(Ref)		1(Ref)		1(Ref)		1(Ref)	
< 30 min	3350	1049 (31.3)	1.23 (1.11 ~ 1.37)	< 0.001	1.18 (1.06 ~ 1.31)	0.002	1.15 (1.02 ~ 1.28)	0.018	1.15 (1.03 ~ 1.29)	0.014
≥ 90 min	872	223 (25.6)	0.91 (0.78 ~ 1.06)	0.214	0.93 (0.8 ~ 1.09)	0.359	0.92 (0.78 ~ 1.09)	0.326	0.93 (0.78 ~ 1.09)	0.365
*P* for trend				0.766		0.758		0.966		0.96

Per hours of TSD, NSD and per 10 min increased of MND as continuous variable. Reference categories are defined as: "no physical dysfunction" (physical dysfunction), "6–8 h" (TSD/NSD), and "30–90 min" (MND)

*PD* physical dysfunction, *TSD* total sleep duration, *NSD* nighttime sleep duration, *MND* midday nap duration, *IDS* incident depression symptoms, *HR* hazard ratio, *CI* confidence interval, *NPD* non-physical dysfunction

^a^Adjusted for sex, age

^b^Adjusted for sex, age, marital status, residence, educational background, alcohol consumption, smoking status, BMI, waist circumference

^c^Adjusted for sex, age, marital status, residence, educational background, alcohol consumption, smoking status, BMI, waist circumference, hypertension, heart disease, stroke, lung disease

**Fig. 2:**
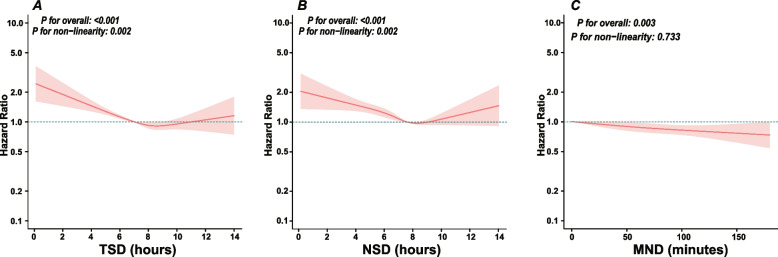
Restricted cubic spline curves for incident depressive symptoms (IDS) by sleep duration after covariate adjustment. **A** Total sleep duration (TSD); **B** nighttime sleep duration (NSD); **C** midday nap duration (MND). Abbreviations: IDS, incident depressive symptoms; TSD, total sleep duration; NSD, nighttime sleep duration; MND, midday nap duration

Restricted cubic splines (Fig. 2. A-C) showed non-linear correlations between TSD, NSD and depression risk (*P* for non-linearity: 0.002), while MND had no significant non-linearity (*P* = 0.733). Threshold analysis (Table 3) revealed that HR decreased significantly with longer duration when TSD < 8 h and NSD < 7.5 h (both *P* < 0.001), but no significant HR change beyond thresholds.

**Table 3:** The non-linearity relationship between TSD, NSD and IDS

Variable	Threshold	Adjusted HR (95%CI)	*P* value
TSD	< 8	0.864 (0.823,0.907)	< 0.001
	≥ 8	1.016 (0.943,1.095)	0.6694
Likelihood Ratio test			< 0.001
NSD	< 7.5	0.872 (0.834,0.913)	< 0.001
	≥ 7.5	1.025 (0.912,1.152)	0.6832
Likelihood Ratio test			0.014

Adjusted for sex, age, marital status, residence, educational background, alcohol consumption, smoking status, BMI, waist circumference, hypertension, heart disease, stroke, lung disease

*TSD* total sleep duration, *NSD* nighttime sleep duration, *IDS* incident depression symptoms, *HR* hazard ratio, *CI* confidence interval, *BMI* body mass index

A restricted cubic spline (RCS) function was fitted into Cox regression Model 3, with four knots placed at the 5th, 35th, 65th, and 95th percentiles of the exposure distribution, to flexibly model the dose–response relationship. The heavy central solid line represents the adjusted hazard ratio, with the shaded area indicating the 95% confidence interval. The model was adjusted for sex, age, marital status, residence, educational background, alcohol consumption, smoking status, body mass index, waist circumference, hypertension, heart disease, stroke, lung disease.

### Combined association of PD and SD with IDS

Taking NPD + TSD 6–8 h, NPD + NSD 6–8 h, and NPD + MND 30–90 min as references (Table 4), PD paired with any duration of TSD, NSD, or MND showed higher hazard ratios (PD + TSD: HR 1.25–1.78; PD + NSD: HR 1.39–1.80; PD + MND: HR 1.26–1.54; all *P* < 0.05). The NPD + SSD combination also yielded elevated HRs (NPD + TSD: 1.29, NPD + NSD: 1.38, both *P* < 0.05; NPD + MND: 1.13, but P = 0.255). Long sleep duration groups, regardless of PD status, did not show significantly elevated risk compared to the reference in most models.

**Table 4:** Combined association of PD, TSD, NSD, and MND with prevalence of IDS

Variable	Unadjusted		Model1^a^		Model2^b^		Model3^c^	
	**HR(95%CI)**	***P* value**	**HR(95%CI)**	***P* value**	**HR(95%CI)**	***P* value**	**HR(95%CI)**	***P* value**
PD/NPD + TSD (depression cases/person-year)								
NPD + TSD 6–8 h (271/8763)	1(Ref)		1(Ref)		1(Ref)		1(Ref)	
NPD + TSD < 6 h (107/2423)	1.5 (1.2 ~ 1.87)	< 0.001	1.45 (1.16 ~ 1.82)	0.001	1.3 (1.04 ~ 1.63)	0.021	1.29 (1.03 ~ 1.62)	0.025
NPD + TSD ≥ 8 h (325/10796)	0.97 (0.83 ~ 1.14)	0.716	0.98 (0.83 ~ 1.15)	0.783	0.94 (0.8 ~ 1.11)	0.461	0.94 (0.8 ~ 1.11)	0.476
PD + TSD 6–8 h (419/8337)	1.66 (1.43 ~ 1.94)	< 0.001	1.52 (1.3 ~ 1.77)	< 0.001	1.41 (1.21 ~ 1.64)	< 0.001	1.37 (1.17 ~ 1.61)	< 0.001
PD + TSD < 6 h (244/3491)	2.39 (2.01 ~ 2.84)	< 0.001	2.1 (1.76 ~ 2.51)	< 0.001	1.82 (1.52 ~ 2.17)	< 0.001	1.78 (1.49 ~ 2.13)	< 0.001
PD + TSD ≥ 8 h (449/9456)	1.56 (1.34 ~ 1.81)	< 0.001	1.44 (1.23 ~ 1.67)	< 0.001	1.28 (1.1 ~ 1.49)	0.002	1.25 (1.07 ~ 1.46)	0.004
*P* for trend		< 0.001		< 0.001		< 0.001		< 0.001
PD/NPD + NSD (depression cases/person-year)								
NPD + NSD 6–8 h (300/10604)	1(Ref)		1(Ref)		1(Ref)		1(Ref)	
NPD + NSD < 6 h (139/3290)	1.58 (1.29 ~ 1.93)	< 0.001	1.55 (1.27 ~ 1.89)	< 0.001	1.4 (1.14 ~ 1.71)	0.001	1.38 (1.13 ~ 1.7)	0.002
NPD + NSD ≥ 8 h (264/8088)	1.19 (1.01 ~ 1.4)	0.043	1.18 (1 ~ 1.39)	0.052	1.11 (0.94 ~ 1.31)	0.215	1.1 (0.94 ~ 1.31)	0.211
PD + NSD 6–8 h (501/10022)	1.82 (1.57 ~ 2.1)	< 0.001	1.65 (1.42 ~ 1.9)	< 0.001	1.52 (1.32 ~ 1.76)	< 0.001	1.48 (1.28 ~ 1.71)	< 0.001
PD + NSD < 6 h (276/4346)	2.38 (2.02 ~ 2.8)	< 0.001	2.12 (1.8 ~ 2.51)	< 0.001	1.83 (1.55 ~ 2.17)	< 0.001	1.8 (1.52 ~ 2.13)	< 0.001
PD + NSD ≥ 8 h (335/6916)	1.79 (1.53 ~ 2.09)	< 0.001	1.63 (1.39 ~ 1.91)	< 0.001	1.43 (1.22 ~ 1.67)	< 0.001	1.39 (1.19 ~ 1.63)	< 0.001
*P* for trend		< 0.001		< 0.001		< 0.001		< 0.001
PD/NPD + MND (depression cases/person-year)								
NPD + MND 30–90 min (213/7165)	1(Ref)		1(Ref)		1(Ref)		1(Ref)	
NPD + MND < 30 min (406/11648)	1.22 (1.03 ~ 1.44)	0.018	1.17 (0.99 ~ 1.38)	0.061	1.1 (0.93 ~ 1.31)	0.252	1.13 (0.93 ~ 1.31)	0.255
NPD + MND ≥ 90 min (84/3169)	0.87 (0.68 ~ 1.12)	0.282	0.89 (0.69 ~ 1.15)	0.369	0.88 (0.68 ~ 1.13)	0.311	0.87 (0.68 ~ 1.13)	0.306
PD + MND 30–90 min (330/6803)	1.64 (1.38 ~ 1.95)	< 0.001	1.49 (1.25 ~ 1.77)	< 0.001	1.35 (1.13 ~ 1.61)	0.001	1.31 (1.1 ~ 1.56)	0.003
PD + MND < 30 min (643/11408)	2.03 (1.74 ~ 2.37)	< 0.001	1.77 (1.51 ~ 2.08)	< 0.001	1.58 (1.35 ~ 1.86)	< 0.001	1.54 (1.31 ~ 1.81)	< 0.001
PD + MND ≥ 90 min (139/3073)	1.52 (1.23 ~ 1.88)	< 0.001	1.42 (1.14 ~ 1.76)	0.002	1.28 (1.03 ~ 1.59)	0.026	1.26 (1.01 ~ 1.57)	0.037
*P* for trend		< 0.001		< 0.001		< 0.001		< 0.001

Reference categories are defined as "NPD + TSD6-8 h" (PD/NPD + TSD), "NPD + NSD6-8 h" (PD/NPD + NSD), and "NPD + MND30-90 min" (PD/NPD + MND)

*PD* physical dysfunction, *NPD* no physical dysfunction, *TSD* total sleep duration, *NSD* nighttime sleep duration, *MND* midday nap duration, *IDS* incident depression symptoms, *MSD* moderate sleep duration, *HR* hazard ratio, *CI* confidence interval, *BMI* body mass index

^a^Adjusted for sex, age

^b^Adjusted for sex, age, marital status, residence, educational background, alcohol consumption, smoking status, BMI, waist circumference

^c^Adjusted for sex, age, marital status, residence, educational background, alcohol consumption, smoking status, BMI, waist circumference, hypertension, heart disease, stroke, lung disease

Taking PD + TSD < 6 h, PD + NSD < 6hurs, and PD + MND < 30 min as references (Table 5), HRs of other combinations were lower (other PD combinations: full adjusted HR = 0.70 ~ 0.85, all NPD combinations: full adjusted HR = 0.53 ~ 0.77). *P* for trend was < 0.05 in all models.

**Table 5:** Combined association of PD, TSD, NSD, and MND with prevalence of IDS

Variable	Unadjusted		Model1^a^		Model2^b^		Model3^c^	
	**HR(95%CI)**	***P* value**	**HR(95%CI)**	***P* value**	**HR(95%CI)**	***P* value**	**HR(95%CI)**	***P* value**
PD/NPD + TSD (depression cases/person-year)								
PD + TSD < 6 h (244/3491)	1(Ref)		1(Ref)		1(Ref)		1(Ref)	
PD + TSD 6–8 h (419/8337)	0.7 (0.59 ~ 0.81)	< 0.001	0.72 (0.62 ~ 0.85)	< 0.001	0.78 (0.66 ~ 0.91)	0.002	0.77 (0.66 ~ 0.9)	0.001
PD + TSD ≥ 8 h (449/9456)	0.65 (0.56 ~ 0.76)	< 0.001	0.68 (0.58 ~ 0.8)	< 0.001	0.71 (0.6 ~ 0.83)	< 0.001	0.7 (0.6 ~ 0.82)	< 0.001
NPD + TSD < 6 h (107/2423)	0.63 (0.5 ~ 0.79)	< 0.001	0.69 (0.55 ~ 0.87)	0.002	0.72 (0.57 ~ 0.9)	0.005	0.73 (0.58 ~ 0.91)	0.007
NPD + TSD 6–8 h (271/8763)	0.42 (0.35 ~ 0.5)	< 0.001	0.48 (0.4 ~ 0.57)	< 0.001	0.55 (0.46 ~ 0.66)	< 0.001	0.56 (0.47 ~ 0.67)	< 0.001
NPD + TSD ≥ 8 h (325/10796)	0.41 (0.34 ~ 0.48)	< 0.001	0.47 (0.39 ~ 0.55)	< 0.001	0.52 (0.44 ~ 0.61)	< 0.001	0.53 (0.45 ~ 0.63)	< 0.001
*P* for trend		< 0.001		< 0.001		< 0.001		< 0.001
PD/NPD + NSD (depression cases/person-year)								
PD + NSD < 6 h (276/4346)	1(Ref)		1(Ref)		1(Ref)		1(Ref)	
PD + NSD 6–8 h (501/10022)	0.76 (0.66 ~ 0.88)	< 0.001	0.77 (0.67 ~ 0.9)	0.001	0.83 (0.72 ~ 0.96)	0.013	0.82 (0.71 ~ 0.95)	0.01
PD + NSD ≥ 8 h (335/6916)	0.75 (0.64 ~ 0.88)	< 0.001	0.77 (0.65 ~ 0.9)	0.001	0.78 (0.66 ~ 0.91)	0.002	0.77 (0.66 ~ 0.91)	0.002
NPD + NSD < 6 h (139/3290)	0.66 (0.54 ~ 0.81)	< 0.001	0.73 (0.59 ~ 0.9)	0.003	0.76 (0.62 ~ 0.94)	0.01	0.77 (0.63 ~ 0.95)	0.013
NPD + NSD 6–8 h (300/10604)	0.42 (0.36 ~ 0.49)	< 0.001	0.47 (0.4 ~ 0.56)	< 0.001	0.55 (0.46 ~ 0.65)	< 0.001	0.56 (0.47 ~ 0.66)	< 0.001
NPD + NSD ≥ 8 h (264/8088)	0.5 (0.42 ~ 0.59)	< 0.001	0.55 (0.47 ~ 0.66)	< 0.001	0.61 (0.51 ~ 0.72)	< 0.001	0.62 (0.52 ~ 0.74)	< 0.001
*P* for trend		< 0.001		< 0.001		< 0.001		< 0.001
PD/NPD + MND (depression cases/person-year)								
PD + MND < 30 min (643/11408)	1(Ref)		1(Ref)		1(Ref)		1(Ref)	
PD + MND 30–90 min (330/6803)	0.81 (0.71 ~ 0.92)	0.002	0.84 (0.73 ~ 0.96)	0.01	0.85 (0.75 ~ 0.98)	0.02	0.85 (0.74 ~ 0.97)	0.017
PD + MND ≥ 90 min (139/3073)	0.75 (0.62 ~ 0.9)	0.002	0.8 (0.66 ~ 0.96)	0.016	0.81 (0.67 ~ 0.97)	0.025	0.82 (0.68 ~ 0.99)	0.034
NPD + MND < 30 min (406/11648)	0.6 (0.53 ~ 0.68)	< 0.001	0.66 (0.58 ~ 0.75)	< 0.001	0.7 (0.61 ~ 0.79)	< 0.001	0.71 (0.63 ~ 0.81)	< 0.001
NPD + MND 30–90 min (213/7165)	0.49 (0.42 ~ 0.57)	< 0.001	0.56 (0.48 ~ 0.66)	< 0.001	0.63 (0.54 ~ 0.74)	< 0.001	0.65 (0.55 ~ 0.76)	< 0.001
NPD + MND ≥ 90 min (84/3169)	0.43 (0.34 ~ 0.54)	< 0.001	0.5 (0.4 ~ 0.63)	< 0.001	0.55 (0.44 ~ 0.7)	< 0.001	0.57 (0.45 ~ 0.72)	< 0.001
*P* for trend		< 0.001		< 0.001		< 0.001		< 0.001

Reference categories are defined as "PD + TSD < 6 h" (PD/NPD + TSD), "PD + NSD < 6 h" (PD/NPD + NSD), and "PD + MND < 30 min" (PD/NPD + MND)

*PD* physical functioning, *NPD* non-physical functioning, *TSD* total sleep duration, *NSD* nighttime sleep duration, *MND* midday nap duration, *IDS* incident depression symptom, *SSD* short sleep duration, *HR* hazard ratio, *CI* confidence interval, *BMI* body mass index

^a^Adjusted for sex, age;

^b^Adjusted for sex, age, marital status, residence, educational background, alcohol consumption, smoking status, BMI, waist circumference;

^c^Adjusted for sex, age, marital status, residence, educational background, alcohol consumption, smoking status, BMI, waist circumference, hypertension, heart disease, stroke, lung disease

### Subgroup and sensitivity analysis

Forest plot displaying the adjusted HR and 95% CI for incident depressive symptoms (IDS) across various subgroups. The analysis was conducted using a multivariable Cox regression model, adjusting for sex, age, marital status, residence, educational background, alcohol consumption, smoking status, BMI, waist circumference, hypertension, heart disease, stroke, lung disease. Subgroup analyses by age, sex, physical function, and presence of hypertension etc. are shown. The *P*-values for interaction indicate no significant differences in the effect of IDS across subgroups (*P* for interaction > 0.05 for all).

**Fig. 3:**
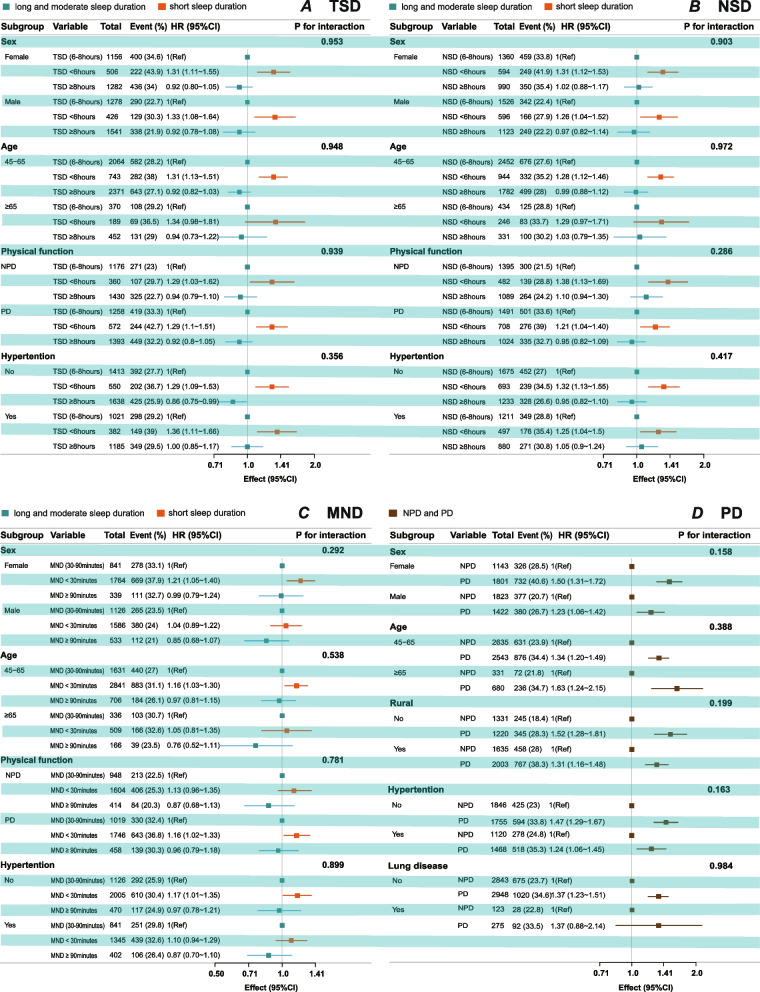
Subgroup analyses of the associations of sleep duration and physical function with incident depressive symptoms (IDS) using multivariable Cox regression models. **A** Total sleep duration (TSD); **B** nighttime sleep duration (NSD); **C** midday nap duration (MND); **D** physical dysfunction (PD) versus no physical dysfunction (NPD). Abbreviations: PD, physical dysfunction; NPD, no physical dysfunction; TSD, total sleep duration; NSD, nighttime sleep duration; MND, midday nap duration; HR, hazard ratio; CI, confidence interval; BMI, body mass index

Using moderate sleep duration of TSD, NSD, MND and NPD as reference, subgroup analyses of independent PD, TSD, NSD and MND with IDS (Fig. 3) showed trends consistent with Table 2 across all strata (sex, age, hypertension, etc.), and no significant interactions were detected (all P for interaction > 0.05). In panels A, B and C(both PD/NPD strata) SSD’s 95% CI lay entirely to the right of the null line, but the CI crossed the null in NPD subgroup, whereas LSD remained non-significant in all three panels.

To reduce age-related heterogeneity in PF, SD and depressive presentations, we also divided age into seven 5-year intervals for subgroup analysis (Table S1−4). Among participants aged 45–50 years, the LSD category (≥ 8 h) within both TSD and NSD strata yielded HRs of 0.64 and 0.70, respectively (both P < 0.05), diverging from other age groups. In the ≥ 75 years NSD stratum, the SSD category (≤ 6 h) showed an HR of 0.19 (*P* = 0.02).

In the sensitivity analysis of the independent and joint associations of PD and SD with IDS, we repeated the multivariable regression models, using the dataset prior to multiple imputation (Table S5-S7). Moreover, after removing items of CES-D scale related to sleep and physical functioning (Table S8), and when PD employed as a continuous variable according to questionnaire scores and subsequently grouped by the 25th, 50th, and 75th percentiles, with cut-off values of 1, 2, and 4, respectively(Table S9), we reperformed the multivariable regression analyses. All findings remained essentially unchanged.

### The interaction effects between PD and SD on DS

Interaction analyses (Table S10-S11) showed that, for both SSD and LSD, the multiplicative interaction with PD was not statistically significant (full adjusted P values on the multiplicative scale > 0.05). The 95% confidence intervals of RERI and AP all included zero, and the 95% CI of SI included one, indicating no significant additive interaction between PD and SD in the risk of DS.

### The mediating effects between PD and SD on DS

**Fig. 4:**
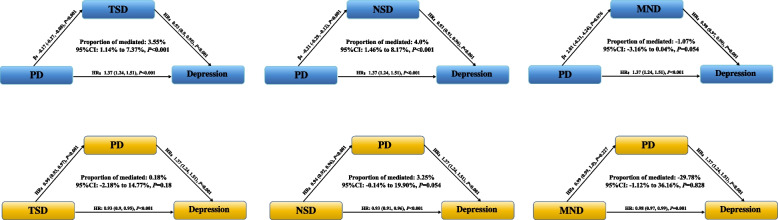
Mediating effects of sleep duration on the association between physical dysfunction and new-onset depression. Abbreviations: PD, physical functioning; TSD, total sleep duration; NSD, nighttime sleep duration; MND, midday nap duration; HR, hazard ratio; CI, confidence interval

Bidirectional mediation analysis was conducted to examine the role of TSD, NSD, and MND in the association between PD and new-onset depression (Fig. 4). TSD and NSD showed significant positive mediation effects, with proportions mediated of 3.55% (95% CI: 1.14%–7.37%) and 4.0% (95% CI: 1.46%–8.17%), respectively (all *P* < 0.001). The mediation effect of MND was not statistically significant (proportion = 3.25%; 95% CI: –0.14%–19.90%; *P* = 0.054). No significant reverse mediation effects were observed (all *P* > 0.05).

## Discussion

Using CHARLS data, we found that both PD and SSD were associated with an elevated risk of IDS, whereas long sleep duration was not associated with an increased risk of IDS. Subgroup analyses revealed heterogeneity by functional status. Among participants NPD, MND was not significantly associated with IDS, whereas SSD in NSD appeared to be more salient. In contrast, among participants with PD, SSD across any sleep dimension was consistently associated with a higher risk of IDS. In the joint-exposure analyses, the “PD + SSD” combination consistently conferred the highest risk. Mediation analyses further suggested that TSD and NSD explained a small proportion of the association between PD and depressive symptoms/IDS, while neither additive nor multiplicative interactions between PD and sleep duration were supported. Collectively, these findings highlight the importance of simultaneously considering functional status and sleep hygiene in depression prevention among aging populations [12].

Prior studies have consistently suggested that physical functional impairment is associated with a higher risk of depression. ADL disability has been shown to be associated with an increased risk of depression among adults aged ≥ 55 years [28]. Previous evidence also indicates that physical dysfunction, greater functional limitations [29], and ADL disability [30] are associated with depression risk. For example, Yumeng Yan and colleagues reported in a rural middle-aged and older Chinese population that PD was associated with an elevated risk of depression and further suggested that PD may predict incident depression [18]. Consistent with this body of evidence, we likewise observed that PD was associated with an increased risk of IDS. Moreover, after incorporating sleep duration into the same analytical framework, the “PD + SSD” combination remained the most consistently high-risk category in the joint grouping. This finding adds public-health value: many prior studies defined functional impairment at a later stage characterized by ADL/IADL disability, or examined only a single physical performance indicator. In contrast, our study focused on an earlier stage of functional decline prior to overt ADL/IADL disability and characterized risk patterns using multidimensional sleep measures (TSD, NSD, and MND), thereby aligning more closely with a prevention paradigm that emphasizes earlier risk identification and an advanced intervention window.

Regarding evidence on sleep duration and IDS, prior meta-analyses have generally concluded that SSD is a risk factor for IDS [12], whereas the direction of association for LSD varies across studies and specific populations; some studies have reported a U-shaped association in which LSD is also linked to higher risk [31, 32]. In our study, we did not observe an independent risk associated with LSD. RCS analyses further suggested an “L-shaped” association of TSD and NSD with IDS: beyond approximately 7.5–8 h of sleep, the risk difference was no longer statistically significant. More importantly, age-stratified subgroup analyses indicated age-related heterogeneity in the sleep–IDS association. In the 45–50-year group, SSD was associated with higher risk, whereas long sleep (≥ 8 h) showed an inverse (protective) association; in the ≥ 75-year group, short sleep showed an inverse association. These age-varying directions suggest that the relationship between sleep duration and IDS may be influenced by age-related changes in physiological sleep need, differences in health status, and lifestyle factors. A more cautious interpretation, therefore, is that these subgroup findings underscore the importance of stratified assessment and individualized sleep management rather than applying a single duration threshold uniformly across all age groups. In addition, another subgroup finding provides more actionable clues for intervention: among individuals without PD, maintaining NSD ≥ 6 h may be a more critical feature for risk reduction; among individuals with PD, NSD ≥ 6 h or MND ≥ 30 min was associated with lower IDS risk. Compared with generic recommendations to “sleep more” or “sleep less” such function-stratified combinations may be more readily translatable into practice.

At the mechanistic level, the associations among PD, SD, and IDS are more likely to operate through intertwined social and behavioral pathways, with sleep duration functioning as a “partial pathway”.Social participation and role-function pathway: PD often entails reduced mobility and restrictions in daily activities, which in turn lower the frequency of going out and social interaction, undermine the fulfillment of family and social roles, and weaken perceived self-efficacy and sense of control [33, 34]. Reduced social participation, role limitation, and heightened loneliness may increase psychological stress and negative affect, thereby increasing susceptibility to IDS [35–37].

Activity–routine–sleep-structure pathway: PD is frequently accompanied by pain, fatigue, or fear of falling, leading individuals to reduce exercise and outdoor activities and to spend more time sedentary. This may weaken daytime activity rhythms and subsequently disrupt circadian regulation and sleep structure (e.g., insufficient nighttime sleep, sleep fragmentation, or “compensatory” daytime sleep/napping through prolonged naps) [38–41]. Within this chain, SD—particularly TSD and NSD—can be viewed as an observable segment through which PD may influence IDS. In our study, TSD and NSD partially mediated the PD–IDS association, suggesting that “functional decline → changes in SD → increased depression risk” may indeed represent one pathway. However, the modest proportion mediated also indicates that SD is unlikely to be the primary or sole mechanism linking PD to IDS, and the potential contribution of sleep quality warrants further attention.

Emotion regulation and stress-coping pathway: Sleep insufficiency can impair emotion regulation, increase negative rumination and sensitivity to adverse events, and reduce stress-recovery capacity, thereby elevating the risk of IDS [42, 43]. Among individuals with PD, fatigue induced by insufficient sleep may compound functional limitations and further diminish emotion-regulation capacity. This may promote further reductions in activity and social engagement, forming a negative cycle of “functional decline → insufficient sleep → emotional vulnerability → further withdrawal” [44–46]. This pathway may help explain why, among participants with PD, short sleep across multiple sleep dimensions was more consistently associated with higher IDS risk.

### Strengths and limitations

This study makes several methodological and translational advances. Methodological strengths: Our exposure variable, PD, integrates assessments of lower-limb, trunk, and upper-limb function, rather than focusing solely on lower-extremity performance. PD was defined as “inability to perform at least one of nine physical tasks independently.” This criterion shifts attention to earlier stages of functional decline—before the onset of ADL disability—allowing sleep interventions to be initiated earlier and, ideally, preventing IDS. For sleep duration, we examined short-, mid-, and long-duration categories of TSD, NSD, and MND in both isolated and combined fashions, across PD and NPD groups, to assess their associations with IDS. Translational strengths: Mediation analyses demonstrated that both TSD and NSD positively mediate the pathway from PD to IDS, supplying mechanistic evidence that sleep duration partly explains how PD elevates depression risk. Subgroup analyses further identified optimal sleep-duration ranges that minimize depression likelihood for both NPD and PD individuals, offering readily implementable prevention strategies.

This study has several limitations. First, both sleep duration and physical function were self-reported, which may be subject to recall bias and misclassification. In particular, we operationalized physical dysfunction using a binary definition as “reporting difficulty in any of the nine physical function items.” This dichotomization may be overly coarse and may fail to capture substantial heterogeneity in the severity of functional limitations, thereby introducing a risk of misclassification bias. Although the sensitivity analyses yielded findings broadly consistent with those based on the primary binary definition (Table S9), the results should still be interpreted with caution. Second, we focused on sleep duration as the primary sleep-related exposure but did not incorporate indicators of sleep quality (e.g., insomnia symptoms, sleep fragmentation, or sleep efficiency). Given the well-established associations of sleep quality with depressive symptoms and physical function, the absence of sleep-quality measures may result in residual confounding and may, to some extent, limit the interpretability of the mediation findings [47, 48]. Third, as an observational cohort study, causal inference cannot be established. In addition, because CHARLS covers only middle-aged and older adults in China, the generalizability of our findings to other sociocultural contexts or ethnic groups may be limited. Future studies are warranted to incorporate objective sleep assessments, adjust for a more comprehensive set of covariates, and further elucidate the underlying mechanisms.

## Conclusions

In conclusion, PD and SSD are independent risk factors for IDS in Chinese middle-aged and older adults, with PD + SSD combination conferring the highest risk. These recommended risk-reduction strategies are: for NPD individuals, ensure NSD ≥ 6 h; for PD individuals, encourage NSD ≥ 6 h or MND ≥ 30 min. Total and nighttime sleep partially mediate the PD-IDS relationship. Long sleep duration did not increase the risk of IDS. No multiplicative or additive interaction was observed. These findings highlight the importance of improving PF and promoting adequate sleep in preventing depression in older adults.

## Supplementary Information

Supplementary Material 1.

## Data Availability

The data of this study are available in China Health and Retirement Longitudinal Study. https://charls.charlsdata.com/pages/data/111/zh-cn.html.

## Abbreviations

CHARLS: China Health and Retirement Longitudinal Study
CES-D: The Center for Epidemiological Studies Depression Scale
PF: Physical function
PD: Physical dysfunction
NPD: No physical dysfunction
TSD: Total sleep duration
NSD: Nighttime sleep duration
MND: Midday nap duration
LSD: Long sleep duration
MSD: Moderate sleep duration
SSD: Short sleep duration
IDS: Incident depression symptoms
HR: Hazard ratio
CI: Confidence interval
BMI: Body mass index
RCS: Restricted cubic spline
RERI: Relative excess risk due to interaction
AP: Attributable proportion of interaction
SI: Synergy index
