# Supplementary material for: Joint association of physical function and sleep duration with incident depressive symptoms among middle-aged and older Chinese
Source: BMC Public Health. 2026 Feb 21;26:1030. doi: 10.1186/s12889-026-26677-x (PMC13032210; doi:10.1186/s12889-026-26677-x)
Supplement: Supplementary file 1 — Supplementary Material 1. [file 12889_2026_26677_MOESM1_ESM.docx]

**Supplementary material**

Table S1.Subgroup analyses for PD - IDS stratified by age using multivariable Cox regression model

Table S2.Subgroup analyses for TSD - IDS stratified by age using multivariable Cox regression model

Table S3.Subgroup analyses for NSD - IDS stratified by age using multivariable Cox regression model

Table S4.Subgroup analyses for MND - IDS stratified by age using multivariable Cox regression model

Table S5. Sensitive analysis of independent PD, TSD, NSD, and MND with IDS

Table S6. Sensitive analysis of combined PD, TSD, NSD, and MND with IDS

Table S7. Combined association of PD, TSD, NSD, and MND with prevalence of incident depression

Table S8. Multivariable Cox analysis of PD, TSD, NSD, and MND with IDS after removing items of CES-D scale related to sleep and physical functioning

Table S9. Multivariable Cox regression analysis of PD score and IDS

Table S10. The interaction between short sleep duration and PD on IDS

Table S11.The interaction between long sleep duration and PD on IDS

**Table S1.Subgroup analyses for PD - IDS stratified by age using multivariable Cox regression model.**

| **Subgroup** | **Variable** | **Total** | **Event %** | **Crude HR (95%CI)** | **Crude *P* value** | **Adjusted HR (95%CI)** | **Adjusted *P* value** | ***P* for interaction** |
| --- | --- | --- | --- | --- | --- | --- | --- | --- |
| Age,years |  |  |  |  |  |  |  | 0.04 |
| 45-50 | NPD | 871 | 180 (20.7) | 1(Ref) |  | 1(Ref) |  |  |
|  | PD | 661 | 223 (33.7) | 1.92 (1.58~2.34) | <0.001 | 1.83 (1.47~2.28) | <0.001 |  |
| 50-55 | NPD | 593 | 155 (26.1) | 1(Ref) |  | 1(Ref) |  |  |
|  | PD | 536 | 182 (34) | 1.44 (1.16~1.79) | 0.001 | 1.31 (1.02~1.67) | 0.032 |  |
| 55-60 | NPD | 741 | 176 (23.8) | 1(Ref) |  | 1(Ref) |  |  |
|  | PD | 743 | 274 (36.9) | 1.77 (1.47~2.14) | <0.001 | 1.48 (1.19~1.84) | <0.001 |  |
| 60-65 | NPD | 430 | 120 (27.9) | 1(Ref) |  | 1(Ref) |  |  |
|  | PD | 603 | 197 (32.7) | 1.3 (1.03~1.63) | 0.025 | 1.07 (0.84~1.36) | 0.587 |  |
| 65-70 | NPD | 222 | 46 (20.7) | 1(Ref) |  | 1(Ref) |  |  |
|  | PD | 350 | 119 (34) | 2.09 (1.48~2.93) | <0.001 | 1.84 (1.26~2.7) | 0.002 |  |
| 70-75 | NPD | 81 | 20 (24.7) | 1(Ref) |  | 1(Ref) |  |  |
|  | PD | 227 | 82 (36.1) | 1.53 (0.94~2.5) | 0.088 | 1.65 (0.93~2.91) | 0.085 |  |
| ≥ 75 | NPD | 28 | 6 (21.4) | 1(Ref) |  | 1(Ref) |  |  |
|  | PD | 103 | 35 (34) | 1.95 (0.82~4.65) | 0.131 | 1.15 (0.35~3.78) | 0.823 |  |

Adjusted for sex, age, marital status, residence, educational background, alcohol consumption, smoking status, BMI, waist circumference, hypertension, heart disease, stroke, lung disease. Abbreviations: PD, physical functioning; NPD, non-physical functioning; IDS, incident depression symptom; HR, hazard ratio; CI, confidence interval; BMI, body mass index.

**Table S2.Subgroup analyses for TSD - IDS stratified by age using multivariable Cox regression model.**

| **Subgroup** | **Variable** | **Total** | **Event %** | **Crude HR (95%CI)** | **Crude P value** | **Adjusted HR (95%CI)** | **Adjusted P value** | **P for interaction** |
| --- | --- | --- | --- | --- | --- | --- | --- | --- |
| Age, years |  |  |  |  |  |  |  | 0.014 |
| 45-50 | TSD(6-8hours) | 576 | 170 (29.5) | 1(Ref) |  | 1(Ref) |  |  |
|  | TSD(< 6hours) | 173 | 60 (34.7) | 1.37 (1.02~1.84) | 0.035 | 1.18 (0.86~1.64) | 0.309 |  |
|  | TSD(≥ 8hours) | 783 | 173 (22.1) | 0.72 (0.58~0.89) | 0.003 | **0.64 (0.51~0.81)** | **<0.001** |  |
| 50-55 | TSD(6-8hours) | 462 | 124 (26.8) | 1(Ref) |  | 1(Ref) |  |  |
|  | TSD(< 6hours) | 159 | 65 (40.9) | 1.74 (1.29~2.35) | <0.001 | 1.28 (0.92~1.79) | 0.138 |  |
|  | TSD(≥ 8hours) | 508 | 148 (29.1) | 1.1 (0.87~1.4) | 0.436 | 1.09 (0.84~1.42) | 0.529 |  |
| 55-60 | TSD(6-8hours) | 605 | 157 (26) | 1(Ref) |  | 1(Ref) |  |  |
|  | TSD(< 6hours) | 243 | 99 (40.7) | 1.8 (1.4~2.31) | <0.001 | 1.46 (1.11~1.91) | 0.007 |  |
|  | TSD(≥ 8hours) | 636 | 194 (30.5) | 1.09 (0.89~1.35) | 0.407 | 1.07 (0.85~1.35) | 0.548 |  |
| 60-65 | TSD(6-8hours) | 421 | 131 (31.1) | 1(Ref) |  | 1(Ref) |  |  |
|  | TSD(< 6hours) | 168 | 58 (34.5) | 1.22 (0.9~1.66) | 0.208 | 1.17 (0.85~1.62) | 0.326 |  |
|  | TSD(≥ 8hours) | 444 | 128 (28.8) | 0.93 (0.73~1.18) | 0.552 | 0.87 (0.67~1.13) | 0.296 |  |
| 65-70 | TSD(6-8hours) | 213 | 59 (27.7) | 1(Ref) |  | 1(Ref) |  |  |
|  | TSD(< 6hours) | 108 | 44 (40.7) | 1.93 (1.3~2.85) | 0.001 | 1.72 (1.13~2.63) | 0.012 |  |
|  | TSD(≥ 8hours) | 251 | 62 (24.7) | 0.88 (0.62~1.26) | 0.48 | 0.85 (0.57~1.25) | 0.409 |  |
| 70-75 | TSD(6-8hours) | 109 | 37 (33.9) | 1(Ref) |  | 1(Ref) |  |  |
|  | TSD(< 6hours) | 56 | 20 (35.7) | 1.2 (0.7~2.07) | 0.507 | 1.68 (0.9~3.12) | 0.104 |  |
|  | TSD(≥ 8hours) | 143 | 45 (31.5) | 0.97 (0.63~1.5) | 0.884 | 1.23 (0.73~2.09) | 0.442 |  |
| ≥ 75 | TSD(6-8hours) | 48 | 12 (25) | 1(Ref) |  | 1(Ref) |  |  |
|  | TSD(< 6hours) | 25 | 5 (20) | 0.76 (0.27~2.16) | 0.606 | 0.39 (0.09~1.67) | 0.206 |  |
|  | TSD(≥ 8hours) | 58 | 24 (41.4) | 1.38 (0.69~2.76) | 0.363 | 1.17 (0.47~2.93) | 0.732 |  |

Adjusted for sex, age, marital status, residence, educational background, alcohol consumption, smoking status, BMI, waist circumference, hypertension, heart disease, stroke, lung disease. Abbreviations: TSD, total sleep duration; IDS, incident depression symptom; HR, hazard ratio; CI, confidence interval; BMI, body mass index.

**Table S3.Subgroup analyses for NSD - IDS stratified by age using multivariable Cox regression model.**

| **Subgroup** | **Variable** | **Total** | **Event %** | **Crude HR (95%CI)** | **Crude P value** | **Adjusted HR (95%CI)** | **Adjusted P value** | **P for interaction** |
| --- | --- | --- | --- | --- | --- | --- | --- | --- |
| Age, years |  |  |  |  |  |  |  | 0.016 |
| 45-50 | NSD(6-8hours) | 714 | 194 (27.2) | 1(Ref) |  | 1(Ref) |  |  |
|  | NSD(< 6hours) | 223 | 74 (33.2) | 1.43 (1.09~1.87) | 0.009 | 1.23 (0.92~1.65) | 0.169 |  |
|  | NSD(≥ 8hours) | 595 | 135 (22.7) | 0.83 (0.67~1.03) | 0.098 | **0.7 (0.55~0.89)** | **0.004** |  |
| 50-55 | NSD(6-8hours) | 551 | 152 (27.6) | 1(Ref) |  | 1(Ref) |  |  |
|  | NSD(< 6hours) | 197 | 75 (38.1) | 1.58 (1.2~2.09) | 0.001 | 1.26 (0.92~1.71) | 0.143 |  |
|  | NSD(≥ 8hours) | 381 | 110 (28.9) | 1.1 (0.86~1.4) | 0.46 | 1.13 (0.86~1.48) | 0.395 |  |
| 55-60 | NSD(6-8hours) | 704 | 193 (27.4) | 1(Ref) |  | 1(Ref) |  |  |
|  | NSD(< 6hours) | 300 | 107 (35.7) | 1.49 (1.18~1.89) | 0.001 | 1.29 (1~1.68) | 0.051 |  |
|  | NSD(≥ 8hours) | 480 | 150 (31.2) | 1.15 (0.93~1.42) | 0.211 | 1.16 (0.92~1.46) | 0.218 |  |
| 60-65 | NSD(6-8hours) | 483 | 137 (28.4) | 1(Ref) |  | 1(Ref) |  |  |
|  | NSD(< 6hours) | 224 | 76 (33.9) | 1.39 (1.05~1.84) | 0.022 | 1.31 (0.98~1.76) | 0.069 |  |
|  | NSD(≥ 8hours) | 326 | 104 (31.9) | 1.21 (0.94~1.56) | 0.141 | 1.1 (0.84~1.44) | 0.491 |  |
| 65-70 | NSD(6-8hours) | 249 | 65 (26.1) | 1(Ref) |  | 1(Ref) |  |  |
|  | NSD(< 6hours) | 141 | 53 (37.6) | 1.82 (1.27~2.63) | 0.001 | 1.67 (1.13~2.46) | 0.01 |  |
|  | NSD(≥ 8hours) | 182 | 47 (25.8) | 1.06 (0.73~1.54) | 0.76 | 0.94 (0.62~1.41) | 0.765 |  |
| 70-75 | NSD(6-8hours) | 128 | 43 (33.6) | 1(Ref) |  | 1(Ref) |  |  |
|  | NSD(< 6hours) | 75 | 25 (33.3) | 1.17 (0.71~1.91) | 0.535 | 1.59 (0.9~2.8) | 0.111 |  |
|  | NSD(≥ 8hours) | 105 | 34 (32.4) | 1.04 (0.66~1.64) | 0.854 | 1.36 (0.78~2.39) | 0.279 |  |
| ≥ 75 | NSD(6-8hours) | 57 | 17 (29.8) | 1(Ref) |  | 1(Ref) |  |  |
|  | NSD(< 6hours) | 30 | 5 (16.7) | 0.61 (0.22~1.65) | 0.328 | **0.19 (0.05~0.77)** | **0.02** |  |
|  | NSD(≥ 8hours) | 44 | 19 (43.2) | 1.37 (0.71~2.65) | 0.343 | 0.56 (0.22~1.43) | 0.225 |  |

Adjusted for sex, age, marital status, residence, educational background, alcohol consumption, smoking status, BMI, waist circumference, hypertension, heart disease, stroke, lung disease. Abbreviations:NSD, nighttime sleep duration; IDS, incident depression symptom; HR, hazard ratio; CI, confidence interval; BMI, body mass index.

**Table S4. Subgroup analyses MND - IDS stratified by age using multivariable Cox regression model.**

| **Subgroup** | **Variable** | **Total** | **Event %** | **Crude HR (95%CI)** | **Crude P value** | **Adjusted HR (95%CI)** | **Adjusted P value** | **P for interaction** |
| --- | --- | --- | --- | --- | --- | --- | --- | --- |
| Age, years |  |  |  |  |  |  |  | 0.781 |
| 45-50 | MND(30-90minutes) | 512 | 120 (23.4) | 1(Ref) |  | 1(Ref) |  |  |
|  | MND(< 30minutes) | 807 | 230 (28.5) | 1.32 (1.05~1.64) | 0.015 | 1.21 (0.95~1.55) | 0.125 |  |
|  | MND(≥ 90minutes) | 213 | 53 (24.9) | 1.09 (0.79~1.51) | 0.592 | 1 (0.7~1.42) | 0.992 |  |
| 50-55 | MND(30-90minutes) | 360 | 100 (27.8) | 1(Ref) |  | 1(Ref) |  |  |
|  | MND(< 30minutes) | 621 | 191 (30.8) | 1.24 (0.97~1.57) | 0.087 | 1.12 (0.85~1.48) | 0.416 |  |
|  | MND(≥ 90minutes) | 148 | 46 (31.1) | 1.14 (0.8~1.61) | 0.468 | 1.27 (0.85~1.87) | 0.241 |  |
| 55-60 | MND(30-90minutes) | 434 | 130 (30) | 1(Ref) |  | 1(Ref) |  |  |
|  | MND(< 30minutes) | 864 | 277 (32.1) | 1.16 (0.94~1.43) | 0.158 | 1.12 (0.89~1.41) | 0.339 |  |
|  | MND(≥ 90minutes) | 186 | 43 (23.1) | 0.71 (0.5~1) | 0.053 | 0.82 (0.57~1.19) | 0.295 |  |
| 60-65 | MND(30-90minutes) | 325 | 90 (27.7) | 1(Ref) |  | 1(Ref) |  |  |
|  | MND(< 30minutes) | 549 | 185 (33.7) | 1.32 (1.02~1.69) | 0.032 | 1.25 (0.96~1.63) | 0.094 |  |
|  | MND(≥ 90minutes) | 159 | 42 (26.4) | 0.94 (0.65~1.35) | 0.728 | 0.94 (0.64~1.38) | 0.743 |  |
| 65-70 | MND(30-90minutes) | 183 | 50 (27.3) | 1(Ref) |  | 1(Ref) |  |  |
|  | MND(< 30minutes) | 290 | 95 (32.8) | 1.42 (1.01~2) | 0.046 | 1.2 (0.83~1.74) | 0.331 |  |
|  | MND(≥ 90minutes) | 99 | 20 (20.2) | 0.7 (0.42~1.17) | 0.176 | 0.75 (0.43~1.31) | 0.31 |  |
| 70-75 | MND(30-90minutes) | 108 | 39 (36.1) | 1(Ref) |  | 1(Ref) |  |  |
|  | MND(< 30minutes) | 154 | 49 (31.8) | 0.79 (0.52~1.2) | 0.273 | 0.72 (0.43~1.21) | 0.212 |  |
|  | MND(≥ 90minutes) | 46 | 14 (30.4) | 0.85 (0.46~1.56) | 0.596 | 0.83 (0.4~1.73) | 0.621 |  |
| ≥ 75 | MND(30-90minutes) | 45 | 14 (31.1) | 1(Ref) |  | 1(Ref) |  |  |
|  | MND(< 30minutes) | 65 | 22 (33.8) | 1 (0.51~1.97) | 0.994 | 0.8 (0.33~1.93) | 0.615 |  |
|  | MND(≥ 90minutes) | 21 | 5 (23.8) | 0.62 (0.22~1.72) | 0.36 | 0.74 (0.19~2.91) | 0.671 |  |

Adjusted for sex, age, marital status, residence, educational background, alcohol consumption, smoking status, BMI, waist circumference, hypertension, heart disease, stroke, lung disease. Abbreviations:MND, midday nap duration; IDS, incident depression symptom; HR, hazard ratio; CI, confidence interval; BMI, body mass index.

**Table S5. Sensitive analysis of independent PD, TSD, NSD, and MND with IDS.**

| **Variable** | **Number** | | **Unadjusted** | | **Model1^a^** | | **Model2^b^** | | **Model3^c^** | |
| --- | --- | --- | --- | --- | --- | --- | --- | --- | --- | --- |
|  | **Total** | **Event(%)** | **HR(95%CI)** | ***P* value** | **HR(95%CI)** | ***P* value** | **HR(95%CI)** | ***P* value** | **HR(95%CI)** | ***P* value** |
| **PD** |  |  |  |  |  |  |  |  |  |  |
| No | 2966 | 703 (23.7) | 1(Ref) |  | 1(Ref) |  | 1(Ref) |  | 1(Ref) |  |
| Yes | 3223 | 1112 (34.5) | 1.67 (1.52~1.83) | <0.001 | 1.51 (1.37~1.66) | <0.001 | 1.48 (1.33~1.65) | <0.001 | 1.46 (1.31~1.62) | <0.001 |
| **TSD** | 6189 | 1815 (29.3) | 0.91 (0.89~0.93) | <0.001 | 0.92 (0.9~0.95) | <0.001 | 0.93 (0.9~0.95) | <0.001 | 0.93 (0.9~0.95) | <0.001 |
| 6-8 hours | 2434 | 690 (28.3) | 1(Ref) |  | 1(Ref) |  | 1(Ref) |  | 1(Ref) |  |
| < 6hours | 932 | 351 (37.7) | 1.53 (1.35~1.74) | <0.001 | 1.45 (1.27~1.65) | <0.001 | 1.35 (1.17~1.54) | <0.001 | 1.34 (1.17~1.54) | <0.001 |
| ≥ 8hours | 2823 | 774 (27.4) | 0.94 (0.85~1.04) | 0.254 | 0.95 (0.86~1.06) | 0.361 | 0.9 (0.81~1.01) | 0.071 | 0.9 (0.81~1.01) | 0.076 |
| *P* for trend |  |  |  | 0.179 |  | 0.288 |  | 0.052 |  | 0.057 |
| **NSD** | 6189 | 1815 (29.3) | 0.92 (0.89~0.95) | <0.001 | 0.93 (0.9~0.96) | <0.001 | 0.93 (0.9~0.96) | <0.001 | 0.93 (0.9~0.96) | <0.001 |
| 6-8 hours | 2886 | 801 (27.8) | 1(Ref) |  | 1(Ref) |  | 1(Ref) |  | 1(Ref) |  |
| < 6hours | 1190 | 415 (34.9) | 1.46 (1.3~1.65) | <0.001 | 1.41 (1.25~1.59) | <0.001 | 1.32 (1.17~1.5) | <0.001 | 1.31 (1.16~1.49) | <0.001 |
| ≥ 8hours | 2113 | 599 (28.3) | 1.05 (0.95~1.17) | 0.358 | 1.05 (0.95~1.17) | 0.328 | 0.99 (0.89~1.11) | 0.917 | 0.99 (0.89~1.11) | 0.903 |
| *P* for trend |  |  |  | 0.181 |  | 0.17 |  | 0.857 |  | 0.876 |
| **MND** | 6189 | 1815 (29.3) | 0.97 (0.96~0.98) | <0.001 | 0.98 (0.97~0.99) | <0.001 | 0.98 (0.97~0.99) | 0.001 | 0.98 (0.97~0.99) | 0.001 |
| 30-90 minutes | 1967 | 543 (27.6) | 1(Ref) |  | 1(Ref) |  | 1(Ref) |  | 1(Ref) |  |
| < 30 minutes | 3350 | 1049 (31.3) | 1.23 (1.11~1.37) | <0.001 | 1.18 (1.06~1.31) | 0.002 | 1.15 (1.02~1.28) | 0.018 | 1.15 (1.03~1.29) | 0.014 |
| ≥ 90 minutes | 872 | 223 (25.6) | 0.91 (0.78~1.06) | 0.214 | 0.93 (0.8~1.09) | 0.359 | 0.92 (0.78~1.09) | 0.326 | 0.93 (0.78~1.09) | 0.365 |
| *P* for trend |  |  |  | 0.766 |  | 0.758 |  | 0.966 |  | 0.96 |

Per hours of TSD, NSD and per 10 minutes increased of MND as continuous variable. The table presents a sensitivity analysis using the data before multiple imputation.

a: Adjusted for sex, age;

b: Adjusted for sex, age, marital status, residence, educational background, alcohol consumption, smoking status, BMI, waist circumference;

c: Adjusted for sex, age, marital status, residence, educational background, alcohol consumption, smoking status, BMI, waist circumference, hypertension, heart disease, stroke, lung disease.

Reference categories are defined as: "no physical dysfunction" (physical dysfunction), "6–8 h" (TSD/NSD), and "30–90 min" (MND).Abbreviations: PD, physical dysfunction; TSD, total sleep duration; NSD, ; MND, midday nap duration; IDS, incident depression symptoms; HR, hazard ratio; CI, confidence interval; BMI, body mass index.

**Table S6. Sensitive analysis of combined PD, TSD, NSD, and MND with IDS.**

| **Variable** | **Unadjusted** | | **Model1^a^** | | **Model2^b^** | | **Model3^c^** | |
| --- | --- | --- | --- | --- | --- | --- | --- | --- |
|  | **HR(95%CI)** | ***P* value** | **HR(95%CI)** | ***P* value** | **HR(95%CI)** | ***P* value** | **HR(95%CI)** | ***P* value** |
| PD/NPD+TSD (depression cases/person-year) | | | | | | | | |
| PD + TSD < 6hours (156/3491) | 1(Ref) |  | 1(Ref) |  | 1(Ref) |  | 1(Ref) |  |
| PD + TSD 6-8hours (260/8337) | 0.68 (0.56~0.83) | <0.001 | 0.71 (0.58~0.87) | 0.001 | 0.77 (0.63~0.94) | 0.01 | 0.77 (0.63~0.94) | 0.009 |
| PD + TSD ≥ 8hours (267/9456) | 0.61 (0.5~0.75) | <0.001 | 0.64 (0.53~0.78) | <0.001 | 0.67 (0.55~0.82) | <0.001 | 0.66 (0.54~0.81) | <0.001 |
| NPD + TSD < 6hours (71/2423) | 0.66 (0.49~0.87) | 0.003 | 0.73 (0.55~0.96) | 0.026 | 0.76 (0.57~1.01) | 0.061 | 0.77 (0.58~1.02) | 0.072 |
| NPD + TSD 6-8hours (135/8763) | 0.33 (0.26~0.42) | <0.001 | 0.38 (0.3~0.48) | <0.001 | 0.45 (0.36~0.57) | <0.001 | 0.46 (0.36~0.58) | <0.001 |
| NPD+ TSD ≥ 8hours (188/10796) | 0.38 (0.3~0.47) | <0.001 | 0.43 (0.35~0.54) | <0.001 | 0.49 (0.39~0.61) | <0.001 | 0.5 (0.4~0.62) | <0.001 |
| *P* for trend |  | <0.001 |  | <0.001 |  | <0.001 |  | <0.001 |
| PD/NPD+NSD (depression cases/person-year) | | | | | | | | |
| PD + NSD < 6hours (177/4346) | 1(Ref) |  | 1(Ref) |  | 1(Ref) |  | 1(Ref) |  |
| PD + NSD 6-8hours (302/10022) | 0.73 (0.6~0.87) | 0.001 | 0.74 (0.61~0.89) | 0.001 | 0.8 (0.66~0.96) | 0.016 | 0.79 (0.65~0.95) | 0.013 |
| PD + NSD ≥ 8hours (204/6916) | 0.72 (0.59~0.88) | 0.001 | 0.73 (0.6~0.89) | 0.002 | 0.74 (0.61~0.91) | 0.004 | 0.74 (0.6~0.9) | 0.003 |
| NPD + NSD < 6hours (87/3290) | 0.65 (0.51~0.84) | 0.001 | 0.72 (0.56~0.93) | 0.013 | 0.76 (0.59~0.99) | 0.041 | 0.77 (0.59~1) | 0.047 |
| NPD + NSD 6-8hours (151/10604) | 0.34 (0.27~0.42) | <0.001 | 0.38 (0.3~0.47) | <0.001 | 0.45 (0.36~0.56) | <0.001 | 0.46 (0.36~0.57) | <0.001 |
| NPD + NSD ≥ 8hours (156/8088) | 0.47 (0.38~0.58) | <0.001 | 0.52 (0.42~0.65) | <0.001 | 0.58 (0.46~0.72) | <0.001 | 0.59 (0.47~0.73) | <0.001 |
| *P* for trend |  | <0.001 |  | <0.001 |  | <0.001 |  | <0.001 |
| PD/NPD+MND (depression cases/person-year) | | | | | | | | |
| PD + MND < 30 minutes (414/11408) | 1(Ref) |  | 1(Ref) |  | 1(Ref) |  | 1(Ref) |  |
| PD + MND 30-90 minutes (196/6803) | 0.75 (0.63~0.89) | 0.001 | 0.79 (0.67~0.94) | 0.006 | 0.81 (0.68~0.96) | 0.015 | 0.81 (0.68~0.96) | 0.015 |
| PD + MND ≥ 90 minutes (73/3073) | 0.62 (0.48~0.79) | <0.001 | 0.66 (0.52~0.85) | 0.001 | 0.67 (0.52~0.86) | 0.002 | 0.68 (0.53~0.87) | 0.002 |
| NPD + MND < 30 minutes (228/11648) | 0.53 (0.45~0.62) | <0.001 | 0.58 (0.5~0.69) | <0.001 | 0.62 (0.53~0.73) | <0.001 | 0.64 (0.54~0.75) | <0.001 |
| NPD + MND 30-90 minutes (121/7165) | 0.44 (0.36~0.54) | <0.001 | 0.51 (0.42~0.63) | <0.001 | 0.59 (0.48~0.73) | <0.001 | 0.61 (0.49~0.75) | <0.001 |
| NPD + MND ≥ 90 minutes (45/3169) | 0.37 (0.27~0.5) | <0.001 | 0.44 (0.32~0.59) | <0.001 | 0.48 (0.35~0.66) | <0.001 | 0.5 (0.36~0.68) | <0.001 |
| *P* for trend |  | <0.001 |  | <0.001 |  | <0.001 |  | <0.001 |

a:Adjusted for sex, age;

b:Adjusted for sex, age, marital status, residence, educational background, alcohol consumption, smoking status, BMI, waist circumference;

c: Adjusted for sex, age, marital status, residence, educational background, alcohol consumption, smoking status, BMI, waist circumference, hypertension, heart disease, stroke, lung disease.

Reference categories are defined as "PD+TSD6-8hours" (PD/NPD+TSD), "PD+NSD6-8hours" (PD/NPD+NSD), and "PD+MND30-90minutes" (PD/NPD+MND). Abbreviations: PD, physical functioning; NPD, non-physical functioning; TSD, total sleep duration; NSD, nighttime sleep duration; MND, midday nap duration; BMI, body mass index; HR, hazard ratio; CI, confidence interval.

**Table S7. Combined association of PD, TSD, NSD, and MND with prevalence of incident depression.**

| **Variable** | **Unadjusted** | | **Model1^a^** | | **Model2^b^** | | **Model3^c^** | |
| --- | --- | --- | --- | --- | --- | --- | --- | --- |
|  | **HR(95%CI)** | ***P* value** | **HR(95%CI)** | ***P* value** | **HR(95%CI)** | ***P* value** | **HR(95%CI)** | ***P* value** |
| Depression (cases/person-year) |  |  |  |  |  |  |  |  |
| NPD + TSD 6-8hours (271/8763) | 1(Ref) |  | 1(Ref) |  | 1(Ref) |  | 1(Ref) |  |
| NPD + TSD < 6hours (107/2423) | 1.5 (1.2~1.87) | <0.001 | 1.45 (1.16~1.82) | 0.001 | 1.28 (1.01~1.64) | 0.044 | 1.27 (1~1.62) | 0.053 |
| NPD+ TSD ≥ 8hours (325/10796) | 0.97 (0.83~1.14) | 0.716 | 0.98 (0.83~1.15) | 0.783 | 0.92 (0.77~1.1) | 0.383 | 0.93 (0.78~1.11) | 0.395 |
| PD + TSD 6-8hours (419/8337) | 1.66 (1.43~1.94) | <0.001 | 1.52 (1.3~1.77) | <0.001 | 1.46 (1.23~1.73) | <0.001 | 1.44 (1.22~1.7) | <0.001 |
| PD + TSD < 6hours (244/3491) | 2.39 (2.01~2.84) | <0.001 | 2.1 (1.76~2.51) | <0.001 | 1.96 (1.62~2.37) | <0.001 | 1.92 (1.59~2.33) | <0.001 |
| PD + TSD ≥ 8hours (449/9456) | 1.56 (1.34~1.81) | <0.001 | 1.44 (1.23~1.67) | <0.001 | 1.32 (1.12~1.56) | 0.001 | 1.3 (1.1~1.54) | 0.002 |
| P for trend |  | <0.001 |  | <0.001 |  | <0.001 |  | <0.001 |
| Depression (cases/person-year) |  |  |  |  |  |  |  |  |
| NPD + NSD 6-8hours (300/10604) | 1(Ref) |  | 1(Ref) |  | 1(Ref) |  | 1(Ref) |  |
| NPD + NSD < 6hours (139/3290) | 1.58 (1.29~1.93) | <0.001 | 1.55 (1.27~1.89) | <0.001 | 1.41 (1.13~1.75) | 0.002 | 1.39 (1.11~1.73) | 0.004 |
| NPD + NSD ≥ 8hours (264/8088) | 1.19 (1.01~1.4) | 0.043 | 1.18 (1~1.39) | 0.052 | 1.14 (0.95~1.37) | 0.159 | 1.13 (0.95~1.36) | 0.173 |
| PD + NSD 6-8hours (501/10022) | 1.82 (1.57~2.1) | <0.001 | 1.65 (1.42~1.9) | <0.001 | 1.61 (1.37~1.89) | <0.001 | 1.58 (1.35~1.86) | <0.001 |
| PD + NSD < 6hours (276/4346) | 2.38 (2.02~2.8) | <0.001 | 2.12 (1.8~2.51) | <0.001 | 2.02 (1.69~2.42) | <0.001 | 1.98 (1.65~2.37) | <0.001 |
| PD + NSD ≥ 8hours (335/6916) | 1.79 (1.53~2.09) | <0.001 | 1.63 (1.39~1.91) | <0.001 | 1.5 (1.26~1.78) | <0.001 | 1.47 (1.23~1.75) | <0.001 |
| P for trend |  | <0.001 |  | <0.001 |  | <0.001 |  | <0.001 |
| Depression (cases/person-year) |  |  |  |  |  |  |  |  |
| NPD + MND 30-90 minutes (213/7165) | 1(Ref) |  | 1(Ref) |  | 1(Ref) |  | 1(Ref) |  |
| NPD + MND < 30 minutes (406/11648) | 1.22 (1.03~1.44) | 0.018 | 1.17 (0.99~1.38) | 0.061 | 1.1 (0.91~1.32) | 0.331 | 1.09 (0.91~1.31) | 0.35 |
| NPD + MND ≥ 90 minutes (84/3169) | 0.87 (0.68~1.12) | 0.282 | 0.89 (0.69~1.15) | 0.369 | 0.87 (0.66~1.15) | 0.325 | 0.87 (0.66~1.15) | 0.338 |
| PD + MND 30-90 minutes (330/6803) | 1.64 (1.38~1.95) | <0.001 | 1.49 (1.25~1.77) | <0.001 | 1.39 (1.14~1.68) | 0.001 | 1.36 (1.12~1.65) | 0.002 |
| PD + MND < 30 minutes (643/11408) | 2.03 (1.74~2.37) | <0.001 | 1.77 (1.51~2.08) | <0.001 | 1.69 (1.41~2.01) | <0.001 | 1.66 (1.39~1.98) | <0.001 |
| PD + MND ≥ 90 minutes (139/3073) | 1.52 (1.23~1.88) | <0.001 | 1.42 (1.14~1.76) | 0.002 | 1.33 (1.05~1.68) | 0.017 | 1.31 (1.04~1.65) | 0.024 |
| P for trend |  | <0.001 |  | <0.001 |  | <0.001 |  | <0.001 |

The table presents a sensitivity analysis using the data before multiple imputation.

a:Adjusted for sex, age;

b:Adjusted for sex, age, marital status, residence, educational background, alcohol consumption, smoking status, BMI, waist circumference;

c: Adjusted for sex, age, marital status, residence, educational background, alcohol consumption, smoking status, BMI, waist circumference, hypertension, heart disease, stroke, lung disease.

Reference categories are defined as "PD+TSD6-8hours" (PD/NPD+TSD), "PD+NSD6-8hours" (PD/NPD+NSD), and "PD+MND30-90minutes" (PD/NPD+MND). Abbreviations: PD, physical dysfunction; TSD, total sleep duration; NSD, nighttime sleep duration; MND, midday nap duration; HR, hazard ratio; CI confidence interval; BMI, body mass index.

**Table S8. Multivariable Cox analysis of PD, TSD, NSD, and MND with IDS after removing items of CES-D scale related to sleep and physical functioning.**

| **Variable** | **Number** | **Unadjusted** | | **Model1^a^** | | **Model2^b^** | | **Model3^c^** | |
| --- | --- | --- | --- | --- | --- | --- | --- | --- | --- |
|  | **Total** | **HR(95%CI)** | ***P* value** | **HR(95%CI)** | ***P* value** | **HR(95%CI)** | ***P* value** | **HR(95%CI)** | ***P* value** |
| **PD** |  |  |  |  |  |  |  |  |  |
| No | 2966 | 1(Ref) |  | 1(Ref) |  | 1(Ref) |  | 1(Ref) |  |
| Yes | 3223 | 1.15 (1.1~1.21) | <0.001 | 1.13 (1.07~1.19) | <0.001 | 1.1 (1.04~1.16) | 0.001 | 1.09 (1.02~1.15) | 0.006 |
| **TSD** | 6189 | 0.96 (0.95~0.98) | <0.001 | 0.97 (0.95~0.98) | <0.001 | 0.97 (0.95~0.98) | <0.001 | 0.97 (0.95~0.98) | <0.001 |
| 6-8 hours | 2434 | 1(Ref) |  | 1(Ref) |  | 1(Ref) |  | 1(Ref) |  |
| < 6hours | 932 | 1.2 (1.11~1.3) | <0.001 | 1.19 (1.1~1.28) | <0.001 | 1.15 (1.06~1.25) | 0.001 | 1.15 (1.06~1.25) | 0.001 |
| ≥ 8hours | 2823 | 0.99 (0.93~1.04) | 0.593 | 0.99 (0.93~1.04) | 0.63 | 0.98 (0.92~1.04) | 0.512 | 0.98 (0.92~1.04) | 0.53 |
| *P* for trend |  |  | <0.001 |  | <0.001 |  | 0.001 |  | 0.001 |
| **NSD** | 6189 | 0.97 (0.96~0.99) | 0.001 | 0.97 (0.96~0.99) | 0.002 | 0.97 (0.96~0.99) | 0.003 | 0.97 (0.96~0.99) | 0.004 |
| 6-8 hours | 2886 | 1(Ref) |  | 1(Ref) |  | 1(Ref) |  | 1(Ref) |  |
| < 6hours | 1190 | 1.23 (1.15~1.32) | <0.001 | 1.22 (1.14~1.3) | <0.001 | 1.19 (1.1~1.28) | <0.001 | 1.19 (1.1~1.28) | <0.001 |
| ≥ 8hours | 2113 | 1.07 (1.01~1.13) | 0.026 | 1.07 (1.01~1.13) | 0.027 | 1.04 (0.98~1.11) | 0.168 | 1.04 (0.98~1.11) | 0.179 |
| *P* for trend |  |  | 0.006 |  | 0.012 |  | 0.014 |  | 0.015 |
| **MND** | 6189 | 0.99 (0.98~0.99) | <0.001 | 0.99 (0.98~0.99) | <0.001 | 0.99 (0.98~1) | 0.001 | 0.99 (0.98~1) | 0.001 |
| 30-90 minutes | 1967 | 1(Ref) |  | 1(Ref) |  | 1(Ref) |  | 1(Ref) |  |
| < 30 minutes | 3350 | 1.14 (1.07~1.2) | <0.001 | 1.12 (1.06~1.19) | <0.001 | 1.11 (1.04~1.18) | 0.001 | 1.11 (1.04~1.18) | 0.001 |
| ≥ 90 minutes | 872 | 0.96 (0.89~1.04) | 0.35 | 0.97 (0.9~1.05) | 0.465 | 0.98 (0.9~1.07) | 0.702 | 0.98 (0.9~1.07) | 0.675 |
| *P* for trend |  |  | <0.001 |  | <0.001 |  | <0.001 |  | <0.001 |

Per hours of TSD, NSD and per 10 minutes increased of MND as continuous variable.

a: Adjusted for sex, age;

b: Adjusted for sex, age, marital status, residence, educational background, alcohol consumption, smoking status, BMI, waist circumference;

c: Adjusted for sex, age, marital status, residence, educational background, alcohol consumption, smoking status, BMI, waist circumference, hypertension, heart disease, stroke, lung disease.

Reference categories are defined as: "no physical dysfunction" (physical dysfunction), "6–8 h" (TSD/NSD), and "30–90 min" (MND). Abbreviations: PD, physical dysfunction; TSD, total sleep duration; NSD, ; MND, midday nap duration; IDS, incident depression symptoms; HR, hazard ratio; CI, confidence interval; BMI, body mass index.

**Table S9. Multivariable Cox regression analysis of PD score and IDS.**

| **Variable** | **Number** | | **Unadjusted** | | **Model1^a^** | | **Model2^b^** | | **Model3^c^** | |
| --- | --- | --- | --- | --- | --- | --- | --- | --- | --- | --- |
|  | **Total** | **Event(%)** | **HR(95%CI)** | ***P* value** | **HR(95%CI)** | ***P* value** | **HR(95%CI)** | ***P* value** | **HR(95%CI)** | ***P* value** |
| **PD score** | 6189 | 1815 (29.3) | 1.09 (1.08~1.11) | <0.001 | 1.08 (1.06~1.09) | <0.001 | 1.07 (1.06~1.09) | <0.001 | 1.07 (1.05~1.09) | <0.001 |
| 0 | 2966 | 703 (23.7) | 1(Ref) |  | 1(Ref) |  | 1(Ref) |  | 1(Ref) |  |
| 1 | 909 | 257 (28.3) | 1.25 (1.08~1.44) | 0.003 | 1.2 (1.04~1.39) | 0.011 | 1.22 (1.04~1.43) | 0.012 | 1.22 (1.05~1.43) | 0.011 |
| 2-3 | 1064 | 340 (32) | 1.53 (1.35~1.75) | <0.001 | 1.4 (1.23~1.6) | <0.001 | 1.38 (1.2~1.59) | <0.001 | 1.37 (1.19~1.58) | <0.001 |
| 4-24 | 1250 | 515 (41.2) | 2.16 (1.92~2.42) | <0.001 | 1.91 (1.69~2.15) | <0.001 | 1.8 (1.58~2.04) | <0.001 | 1.76 (1.54~2) | <0.001 |
| *P* for trend |  |  |  | <0.001 |  | <0.001 |  | <0.001 |  | <0.001 |

a: Adjusted for sex, age;

b: Adjusted for sex, age, marital status, residence, educational background, alcohol consumption, smoking status, BMI, waist circumference;

c: Adjusted for sex, age, marital status, residence, educational background, alcohol consumption, smoking status, BMI, waist circumference, hypertension, heart disease, stroke, lung disease.

PD employed as a continuous variable according to questionnaire scores and subsequently grouped by the 25th, 50th, and 75th percentiles, with cut-off values of 1, 2, and 4, respectively. Abbreviations: PD, physical dysfunction; IDS, incident depression symptoms; HR, hazard ratio; CI, confidence interval.

**Table S10. The interaction between short sleep duration and PD on IDS.**

| Variable | Unadjusted | | | | Full adjusted | | | |
| --- | --- | --- | --- | --- | --- | --- | --- | --- |
|  | HR | 95%CI low | 95%CI up | *P* value | HR | 95%CI low | 95%CI up | *P* value |
| PD+TSD |  |  |  |  |  |  |  |  |
| Multiplicative scale | 0.96 | 0.73 | 1.26 | 0.78 | 1 | 0.76 | 1.32 | 1 |
| RERI | 0.23 | -0.22 | 0.68 | 0.16 | 0.13 | -0.26 | 0.51 | 0.26 |
| AP | 0.1 | -0.09 | 0.28 | 0.16 | 0.07 | -0.14 | 0.27 | 0.26 |
| SI | 1.2 | 0.82 | 1.74 | 0.01 | 1.18 | 0.7 | 1.97 | 0.03 |
| PD+NSD |  |  |  |  |  |  |  |  |
| Multiplicative scale | 0.83 | 0.65 | 1.06 | 0.14 | 0.88 | 0.68 | 1.12 | 0.3 |
| RERI | -0.02 | -0.45 | 0.41 | 0.54 | -0.05 | -0.42 | 0.32 | 0.61 |
| AP | -0.01 | -0.19 | 0.17 | 0.46 | -0.03 | -0.23 | 0.17 | 0.39 |
| SI | 0.99 | 0.72 | 1.35 | 0.18 | 0.94 | 0.63 | 1.41 | 0.23 |
| PD+MND |  |  |  |  |  |  |  |  |
| Multiplicative scale | 1.02 | 0.82 | 1.26 | 0.89 | 1.07 | 0.86 | 1.32 | 0.56 |
| RERI | 0.17 | -0.12 | 0.46 | 0.12 | 0.13 | -0.12 | 0.37 | 0.16 |
| AP | 0.08 | -0.06 | 0.23 | 0.12 | 0.08 | -0.08 | 0.24 | 0.16 |
| SI | 1.2 | 0.85 | 1.69 | 0 | 1.31 | 0.69 | 2.5 | 0.01 |

Short sleep duration: TSD < 6hours; NSD < 6hours; MND < 30minutes.

Abbreviations: PD, physical dysfunction; IDS, incident depression symptoms; HR, hazard ratio; CI, confidence interval; TSD, total sleep duration; NSD, ; MND, midday nap duration; RERI, relative excess risk due to interaction; AP, attributable proportion of interaction; SI, synergy index.

**Table S11.The interaction between long sleep duration and PD on IDS.**

| Variable | Unadjusted | | | | Full adjusted | | | |
| --- | --- | --- | --- | --- | --- | --- | --- | --- |
|  | HR | 95%CI low | 95%CI up | *P* value | HR | 95%CI low | 95%CI up | *P* value |
| PD+TSD |  |  |  |  |  |  |  |  |
| Multiplicative scale | 0.95 | 0.78 | 1.15 | 0.57 | 0.95 | 0.78 | 1.15 | 0.57 |
| RERI | -0.16 | -0.4 | 0.07 | 0.91 | -0.11 | -0.33 | 0.1 | 0.85 |
| AP | -0.12 | -0.28 | 0.05 | 0.09 | -0.1 | -0.28 | 0.09 | 0.15 |
| SI | 0.72 | 0.46 | 1.11 | 0.41 | 0.6 | 0.26 | 1.38 | 0.38 |
| PD+NSD |  |  |  |  |  |  |  |  |
| Multiplicative scale | 0.83 | 0.67 | 1.03 | 0.09 | 0.85 | 0.68 | 1.05 | 0.14 |
| RERI | -0.21 | -0.53 | 0.11 | 0.9 | -0.2 | -0.47 | 0.08 | 0.92 |
| AP | -0.12 | -0.3 | 0.06 | 0.1 | -0.14 | -0.34 | 0.06 | 0.08 |
| SI | 0.79 | 0.57 | 1.1 | 0.4 | 0.65 | 0.38 | 1.11 | 0.42 |
| PD+MND |  |  |  |  |  |  |  |  |
| Multiplicative scale | 0.97 | 0.78 | 1.19 | 0.75 | 0.97 | 0.78 | 1.19 | 0.76 |
| RERI | -0.07 | -0.35 | 0.2 | 0.7 | -0.06 | -0.3 | 0.18 | 0.69 |
| AP | -0.05 | -0.22 | 0.13 | 0.3 | -0.05 | -0.24 | 0.14 | 0.31 |
| SI | 0.88 | 0.58 | 1.36 | 0.28 | 0.79 | 0.35 | 1.79 | 0.29 |

Long sleep duration: TSD ≥ 8hours; NSD ≥ 8hours; MND ≥ 90minutes

Abbreviations: PD, physical dysfunction; IDS, incident depression symptoms; HR, hazard ratio; CI confidence interval; TSD, total sleep duration; NSD, ; MND, midday nap duration; RERI, relative excess risk due to interaction; AP, attributable proportion of interaction; SI, synergy index.
